# Developmental toxicity and brain aromatase induction by high genistein concentrations in zebrafish embryos

**DOI:** 10.1080/15376510802563330

**Published:** 2009-06-30

**Authors:** Dong-Jae Kim, Seung-Hyeok Seok, Min-Won Baek, Hui-Young Lee, Yi-Rang Na, Sung-Hoon Park, Hyun-Kyoung Lee, Noton Kumar Dutta, Koichi Kawakami, Jae-Hak Park

**Affiliations:** 1 Department of Laboratory Animal Medicine, College of Veterinary Medicine and BK2 Program for Veterinary Science, Seoul National University, Seoul, Korea; 2 Division of Molecular and Developmental Biology, National Institute of Genetics and Department of Genetics, The Graduate University of Advanced Studies (SOKENDAI), Mishima, Shizuoka, Japan

**Keywords:** Brain aromatase, Developmental toxicity, EGFP, Genistein, Zebrafish embryo

## Abstract

Genistein is a phytoestrogen found at a high level in soybeans. In vitro and in vivo studies showed that high concentrations of genistein caused toxic effects. This study was designed to test the feasibility of zebrafish embryos for evaluating developmental toxicity and estrogenic potential of high genistein concentrations. The zebrafish embryos at 24 h post-fertilization were exposed to genistein (1 × 10^−4^ M, 0.5 × 10^−4^ M, 0.25 × 10^−4^ M) or vehicle (ethanol, 0.1%) for 60 h. Genistein-treated embryos showed decreased heart rates, retarded hatching times, decreased body length, and increased mortality in a dose-dependent manner. After 0.25 × 10^−4^ M genistein treatment, malformations of survived embryos such as pericardial edema, yolk sac edema, and spinal kyphosis were also observed. TUNEL assay results showed apoptotic DNA fragments in brain. This study also confirmed the estrogenic potential of genistein by EGFP expression in the brain of the mosaic reporter zebrafish embryos. This study first demonstrated that high concentrations of genistein caused a teratogenic effect on zebrafish embryos and confirmed the estrogenic potential of genistein in mosaic reporter zebrafish embryos.

## Introduction

Endocrine-active compounds (EACs) threaten ecosystems by disrupting normal endocrine function, which can lead to reproductive failure in humans and wildlife (Guillette et al. 1994; Jobling et al. 2002). EACs include synthetic estrogen, xeno-estrogen, and phytoestrogen. Synthetic estrogens are pharmaceutical chemicals designed to mimic the action of natural estrogens. Xeno-estrogens are defined as environmental and industrial pollutants. Phytoestrogens are a class of compounds found in a variety of plants (Legler et al. 2000; Ingham et al. 2004).

Genistein (4′,5,7-trihydroxyisoflavone) is a phytoestrogen found in high levels in soybeans (Messina et al. 1994). Genistein has demonstrated anti-proliferative and anti-differentiation effects in many neoplastic cells, and it's prophylactic use has been studied with regards to cancer (Kanatani et al. 1993; Wei et al. 1993; Su et al. 2000; Myoung et al. 2003). High concentrations of genistein have been shown to cause apoptosis and necrosis in testis cells (Kumi-Diaka et al. 1999), primary cortical neuron cultures from Spraugue-Dawley rats (Linford et al. 2001), human thymocytes (McCabe and Orrenius 1993), and human lymphoblastoid cells (Morris et al. 1998). In addition, in vivo studies showed that genistein causes developmental and growth toxicity, myelotoxicity, and brain apoptosis (Choi and Lee 2004; Ingham et al. 2004; Guo et al. 2005).

Estrogen replacement therapy (ERT) is widely used to treat diseases such as Alzheimer's disease, osteoporosis, and cardiovascular disease, that are caused by low levels of estrogen (Kuller 1996; Spritzerm and Wender 2007). Isoflavones that have estrogenic-like effects have been viewed as alternatives to estrogen because long-term usage of ERT increases the risk of breast cancer. In this case, a high dose of isoflavones is needed to be useful for a therapeutic effect, owing to their low efficacy, only about 1–2% of that of ERT (Jin et al. 2007).

Recently, zebrafish embryos have become an exceptional model system for assessing the toxicity of chemicals (Fraysse et al. 2006; Kammann et al. 2006). As the development of zebrafish embryos is rapid and the embryos remain transparent throughout most of embryogenesis, we can easily observe abnormal morphologic changes caused by toxicants. Most of all, zebrafish embryos are very sensitive to toxic chemicals. Previously, we have established xenoestrogen-responsive mosaic zebrafish, in which a zebrafish brain aromatase (AroB) promoter-driven enhanced green fluorescent protein (EGFP) reporter gene was introduced to test estrogenic potential of chemicals. Expression of AroB is most prominent in radial glial cells (Menuet et al. 2003, 2005) and its expression is up-regulated by estrogens (Kazeto et al. 2004). In this model, EGFP was used as a reporter gene. In the presence of xenoestrogens, mosaic reporter zebrafish embryos express EGFP in the brain specifically.

This study was designed to test the feasibility of zebrafish embryos for evaluating developmental toxicity and estrogenic potential of high genistein concentrations.

## Materials and methods

### Chemicals

We obtained Genistein (Sigma, St. Louis, MO) from Veterinary Public Health Laboratory (Seoul National University, Korea) and prepared 20 mM Genistein stock solutions in absolute ethanol. Treatment doses were made from dilution of these stock solutions in Ringer's solution.

### Zebrafish maintenance and eggs collection

Adult zebrafish were raised and maintained on a 14:10 h light:dark cycle at 28.5°C, and bred in tanks as described previously (Westerfield 1995). Mature fish were fed twice daily a combination of Freshwater Aquarium Flake food (TetraWerke, Melle, Germany) and live brine shrimp (San Francisco Bay Brand, Inc., Newark, CA). The care and treatment of the animals were conducted in accordance with the guidelines established by the Seoul National University Institutional Animal Care and Use Committee (Approval No. SNU-050418-2). Fertilized eggs were obtained from naturally reproducing adult zebrafish bred in our laboratory as described previously (Westerfield 1995). Eggs were collected, pooled, rinsed, and placed into clean Ringer's solution.

### Effects of genistein on the development of zebrafish embryos

The zebrafish embryos at 24 h post-fertilization were transferred 12-well plate and exposed to 3 ml of genistein (1 × 10^−4^ M, 0.5 × 10^−4^ M, 0.25 × 10^−4^ M) or vehicle (ethanol, 0.1%) for 60 h. Genistein was dissolved in 100% ethanol and the final ethanol concentration for each treatment was 0.1%. Each treatment group had 60 embryos that were divided into six plates. For each treatment, half of the exposure solution was renewed every day. Per-minute heart rates of genistein-treated embryos 30 h after treatment, the number of hatched embryos, and body length at 37 h after treatment were examined under a stereomicroscope (SZ-PT, OLYMPUS, Japan). Sixty hours after genistein treatment, the mortality and malformation of embryos were determined, and live embryos were fixed in 10% phosphate-buffered formalin for histopathological examination.

To determine whether the toxicity observed in genistein-treated embryos was related to the negative effects on heart rate, zebrafish embryos were exposed to beta-adrenergic receptor agonist isoproterenol (Sigma, St. Louis, MO; Vehicle, 1 × 10^−6^ M, 10 × 10^−6^ M, 100 × 10^−6^ M) in the presence of genistein (0.5 × 10^−4^ M). Experimental methods were the same as described above.

### Histopathological Examination

Whole embryos were fixed in 10% phosphate-buffered formalin for 24 h with slight shaking. Fixed embryos were dehydrated in an alcohol-xylene series, and embedded in paraffin wax. From each block, 2 μm sections were prepared and stained with Hematoxylin and eosin (HE) for histopathological examination.

### TUNEL Assay

To test for apoptosis in genistein-treated embryos, TUNEL assay (terminal deoxynucleotide transferase-mediated dUTP nick-end labeling, In Situ Cell Death Detection Kit, PDO, Roche) was carried out according to the kit manufacturer's instruction. Briefly, 2 μm sections from a paraffin wax-embedded block were deparaffinized and permeabilized by incubation in proteinase K solution (20 μg/ml) for 30 min at 37°C. After two rinses in phosphate-buffered saline (PBS), slides were incubated for 60 min at 37°C in labeling solutions consisting of TdT and fluorescein-conjugated deoxynucleotides in buffer. After three PBS washes, the slides were incubated for 30 min at 37°C in converter-POD (anti-fluorescein peroxidase) solution. Excess converter-POD solution was removed by three PBS rinses, and apoptotic DNA fragments were visualized by incubation in DAB substrates.

### Plasmid Construction

A zebrafish brain aromatase-regulated reporter plasmid, pzfAroB-EGFP, was constructed by linking the proximal promoter region of the zebrafish brain aromatase gene with the enhanced green fluorescent protein (EGFP) reporter gene. A proximal promoter region of the zebrafish brain aromatase gene was amplified by PCR from zebrafish genomic DNA using a specific primer set, zfAroB-F (5′-CCGCTCGAGGTTCAAAGCCCTCCCAAATA-3′) and zfAroB-R (5′-CGGGATCCCCGTCCTCAGGCTTCCATCAT-3′) containing a restriction-digested adaptor (underlined) corresponding to XhoI and BamHI sites, respectively (Kazeto et al. 2001; Menuet et al. 2005). The gene amplification reaction conditions were as follows: first denaturation at 94°C for 3 min; 35 cycles of denaturation at 94°C for 30 s, annealing at 58°C for 30 s, and extension at 72°C for 45 s; and final extension at a cycle of 72°C for 7 min. The PCR product double digested by XhoI and BamHI was subcloned upstream of the EGFP gene in the XhoI/BamHI restriction sites of the T2KXIG vector (Kawakami et al. 2004).

### Microinjection of pzfAroB-EGFP and transposase mRNA

Single-cell fertilized zebrafish embryos were microinjected with pzfAroB-EGFP and transposase mRNA. Transposase mRNA was synthesized in vitro using transposase cDNA (Kawakami et al. 2004) and an mMESSAGE mMACHINE SP6 Kit (Ambion Inc.). The embryos were transferred to agarose ramps, and then injected with a DNA/RNA solution containing 25 ng/μl each of pzfAroB-EGFP circular DNA and transposase mRNA using a micropipette secured in a micromanipulator (World Precision Instruments Inc., Sarasota, FL).

### Genistein-induced EGFP expression in mosaic reporter zebrafish embryos

Two hours after fertilization, the injected embryos were transferred to Petri dishes and exposed to genistein (10^−6^ M) or ethanol alone (0.1%) for 4 days. For each treatment, half of the exposure solution was renewed every day. After genistein exposure, the larvae were transferred to Ringer's solution, as described by Westerfield, and monitored for EGFP expression using an Olympus IX70 microscope equipped with a NIBA2 filter (λex = 470–490 nm; λem = 510–550 nm).

### Statistical analysis

The differences between experimental and control groups were assayed for significance by Duncan's Multiple Range Test (SAS ver. 9.1; SAS Institute Inc., Cary, NC, USA). *P*-values < 0.05 were considered significant.

## Results

### Developmental toxicity of genistein on zebrafish embryos

**Figure 1:**
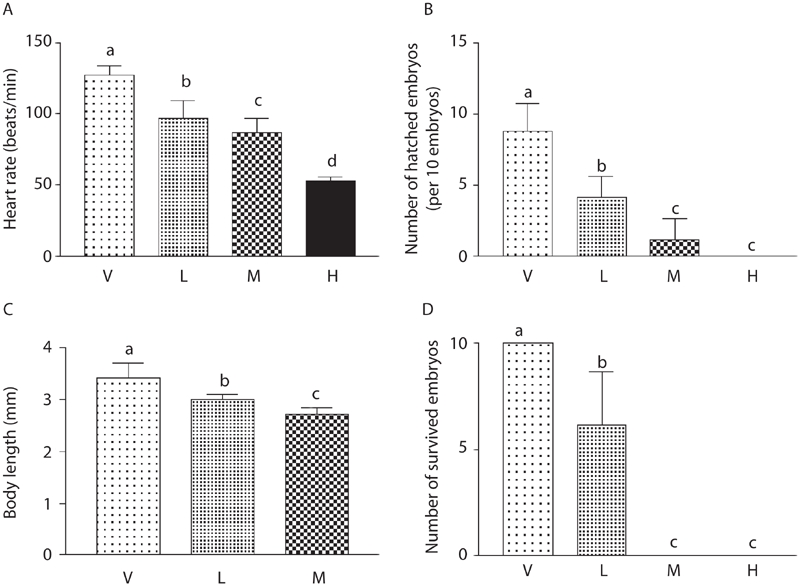
Developmental toxicity of genistein on zebrafish embryos. The zebrafish embryos at 24 h post-fertilization were exposed to genistein (1 × 10^−4^ M, 0.5 × 10^−4^ M, 0.25 × 10^−4^ M) or vehicle (ethanol, 0.1%) for 60 h. (A) Heart rates of genistein-treated embryos at 30 h after treatment decreased in dose-dependent manner. (B) Although about 90% of vehicle-treated embryos hatched at 37 h after treatment, genistein treatment decreased the number of hatched embryos. (C) Body lengths of genistein-treated embryos at 37 h after treatment decreased in a dose-dependent manner. (D) At 60 h after genistein treatment, all embryos survived in the vehicle-treated group, but none of the 0.5 × 10^−4^ M and 1 × 10^−4^ M genistein-treated embryos survived, and about six embryos survived in the 0.25 × 10^−4^ M genistein-treated group [genistein concentration: (V) vehicle, (H) 1 × 10^−4^ M, (M) 0.5 × 10^−4^ M, (L) 0.25 × 10^−4^ M]. Data are expressed as mean ± SD. Values of each group with identical letters in each panel were not significantly different (*p* > 0.05).

**Figure 2:**
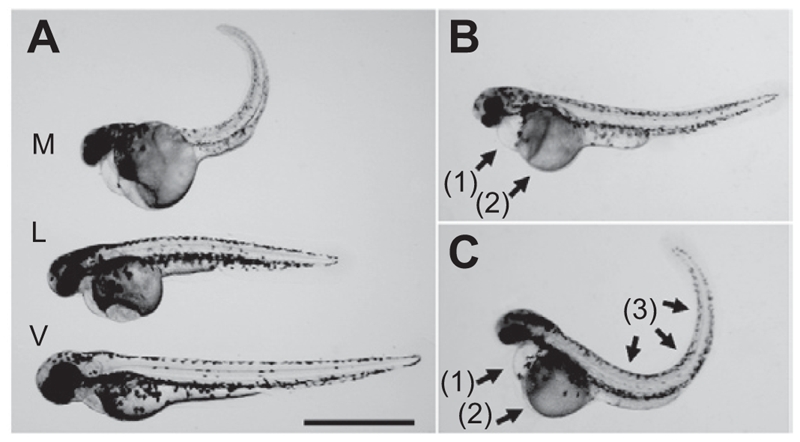
Developmental toxicity of genistein on zebrafish embryos. The zebrafish embryos at 24 h post-fertilization were exposed to genistein or vehicle for 60 h. (A) The body lengths of the genistein-treated embryos decreased in dose-dependent manner [genistein concentration: (V) vehicle, (L) 0.25 × 10^−4^ M, (M) 0.5 × 10^−4^ M]. (B, C) Malformations of embryos that survived after 0.25 × 10^−4^ M genistein treatment such as (1) pericardial edema, (2) yolk sac edema, and (3) spinal kyphosis. Bar = 1 mm.

Per-minute heart rates of genistein-treated embryos 30 h after treatment decreased in a dose-dependent manner (vehicle: 127.62 ± 6.34, 0.25 × 10^−4^ M: 97.12 ± 12.33, 0.5 × 10^−4^ M: 86.87 ± 9.62, 1 × 10^−4^ M: 53.00 ± 2.72 beats/min) (Fig. 1A). Although about 90% of the 10 vehicle-treated embryos in each plate (8.83 ± 1.94 embryos) hatched 37 h after treatment; genistein treatment decreased the number of hatched embryos (0.25 × 10^−4^ M: 4.16 ± 1.47, 0.5 × 10^−4^ M: 1.16 ± 1.47 embryos). None of the ten 1 × 10^−4^ M genistein-treated embryos in each plate hatched (Fig. 1B). The body length of vehicle-treated embryos was 3.43 ± 0.27 mm, but those of genistein-treated embryos decreased in a dose-dependent manner (0.25 × 10^−4^ M: 3.00 ± 0.10, 0.5 × 10^−4^ M: 2.72 ± 0.13 mm) (Fig. 1C and Fig. 2A). Because the embryos treated with the highest concentration of genistein did not hatch, we could not measure the body length of these embryos. Sixty hours after genistein treatment, all embryos survived in the vehicle-treated group, but none of the 0.5 × 10^−4^ M and 1 × 10^−4^ M genistein-treated embryos survived. Moreover, only 6.16 ± 2.48 embryos survived in the 0.25 × 10^−4^ M genistein-treated group (Fig. 1D). We also observed physical malformations in embryos surviving 0.25 × 10^−4^ M genistein treatment such as pericardial edema, yolk sac edema, and spinal kyphosis (Fig. 2B and 2C).

**Figure 3:**
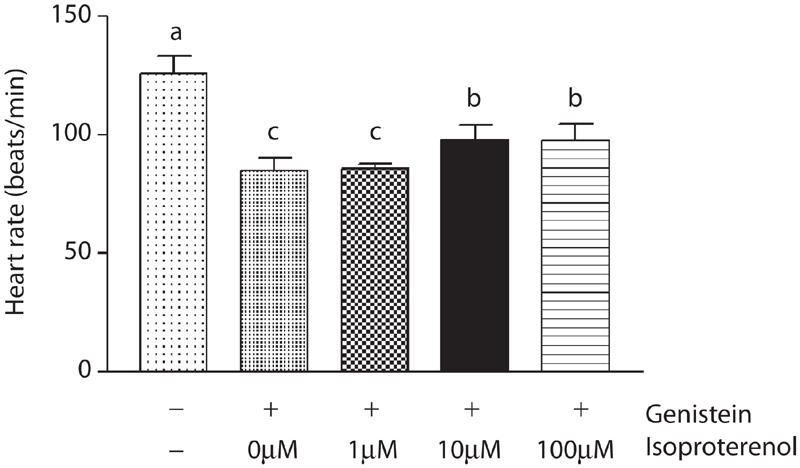
Effect of isoproterenol in the presence of genistein on heart rate in zebrafish embryos. The zebrafish embryos at 24 h post-fertilization were exposed to isoproterenol (Vehicle, 1 × 10^−6^ M, 10 × 10^−6^ M, 100 × 10^−6^ M) in the presence of genistein (0.5 × 10^−4^ M). In the presence of genistein, isoproterenol (10 × 10^−6^ M, 100 × 10^−6^ M) increased heart rate significantly at 30 h after treatment. Data are expressed as mean ± SD. Values of each group with identical letters in each panel were not significantly different (*p* > 0.05).

10 × 10^−6^ M and 100 × 10^−6^ M isoproterenol increased heart rate in the presence of 0.5 × 10^−4^ M genistein (vehicle: 126.00 ± 7.21, genistein only: 85.00 ± 5.56, 1 × 10^−6^ M isoproterenol: 86.00 ± 2.00, 10 × 10^−6^ M isoproterenol: 98.00 ± 6.24, 100 × 10^−6^ M isoproterenol: 97.66 ± 7.02 beats/min) (Fig. 3). However, it could not recover genisterin-induced retarded hatching time, decreased body length, and mortality (data not shown).

### Histopathological examination and TUNEL assay

**Figure 4:**
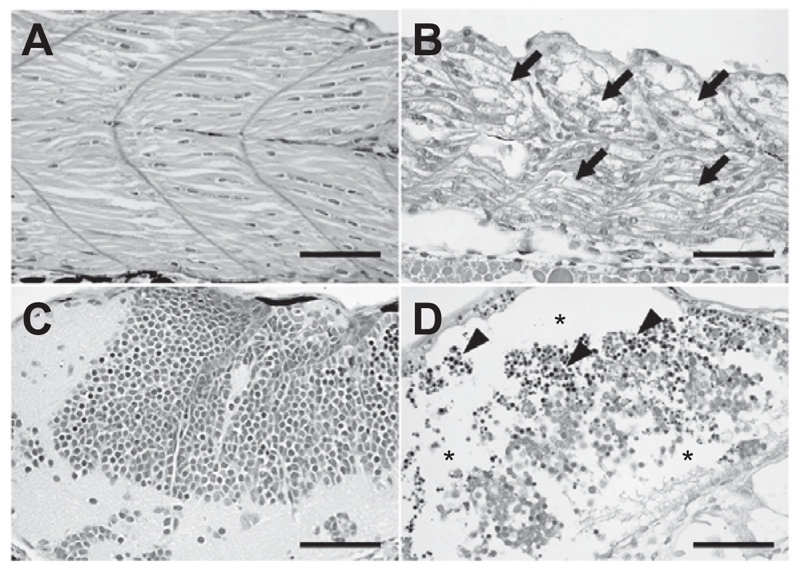
Histopathological examination. The zebrafish embryos at 24 h post-fertilization were exposed to genistein or vehicle for 60 h. The zebrafish embryos that survived after 0.25 × 10^−4^ M genistein or vehicle treatments were prepared for histopathological examination. Histopathological examination revealed that (arrows) granular degeneration of (B) myocytes in skeletal muscle in addition to (asterisks) loss and (arrow heads) apoptosis of (D) neural cells in the brain of the 0.25 × 10^−4^ M genistein-treated group. (A and C) vehicle-treated zebrafish embryos, (B and D) genistein-treated zebrafish embryos. Bar = 50 μm.

**Figure 5:**
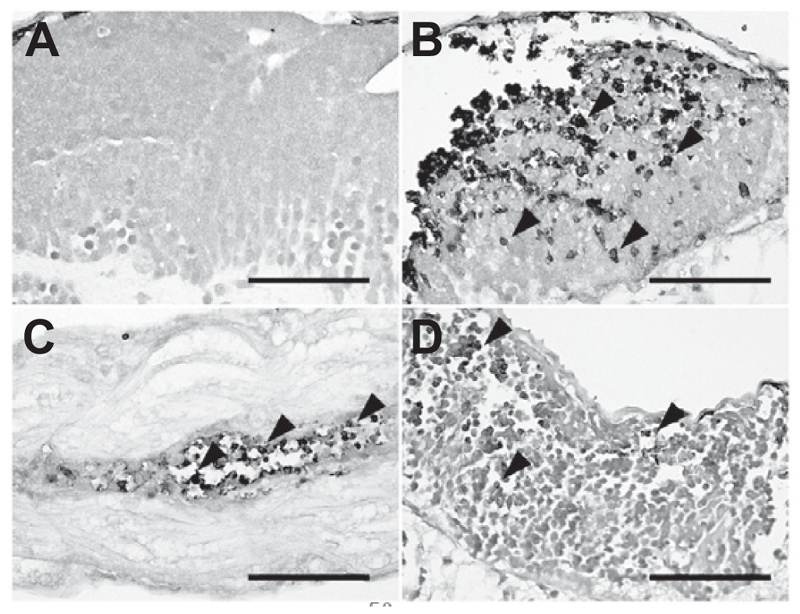
TUNEL assay. To determine whether neural cell damages were related to apoptosis, we conducted a TUNEL assay using the same tissue samples. TUNEL assay results showed apoptotic DNA fragments in (B and D) the brain and (C) spinal cord of 0.25 × 10^−4^ M genistein-treated zebrafish embryos. (A) vehicle treated zebrafish embryos, (B–D) genistein-treated zebrafish embryos. Bar = 50 μm.

Each group of 20 of the zebrafish embryos surviving 0.25 × 10^−4^ M genistein or vehicle treatments were prepared for histopathological examination. Histopathological examination revealed granular degeneration of myocytes in skeletal muscle in addition to loss and apoptosis of neural cells in brain in the all of the 0.25 × 10^−4^ M genistein-treated embryos (Fig. 4). To determine whether neural cell damage was related to apoptosis, we conducted TUNEL analysis using the same tissue samples. TUNEL assay results showed apoptotic DNA fragments in the brain and spinal cord of 0.25 × 10^−4^ M genistein-treated zebrafish embryos (Fig.5).

### AroB reporter gene expression in response to genistein exposure

**Figure 6:**
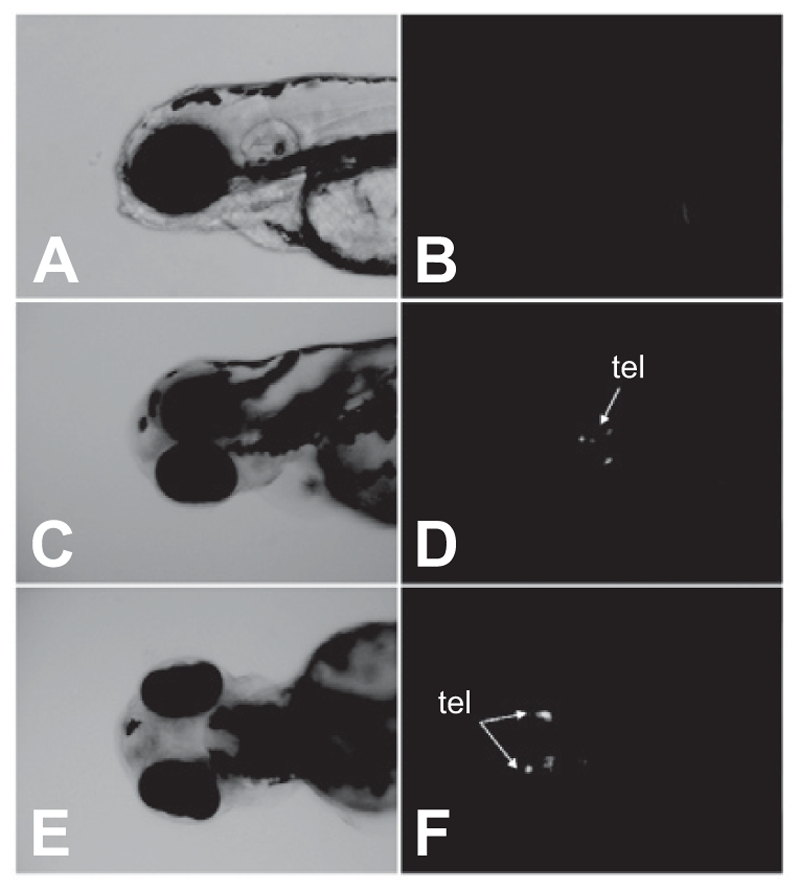
EGFP expression in response to genistein exposure. pzfAroB-EGFP injected embryos were exposed to genistein (10^−6^ M) at 2 hpf for 4 days. EGFP expression was localized in the telencephalon (tel) located in the forebrain. (A and B) vehicle-treated zebrafish embryos, (C–F) genistein-treated zebrafish embryos. (A, C, E: light microscope images, B, D, F: fluorescence microscope images).

To confirm the estrogenic potential of genistein, we used mosaic reporter zebrafish embryos that contained a zebrafish brain aromatase promoter-driven EGFP reporter gene, pzfAr-oB-EGFP. Mosaic reporter zebrafish embryos were exposed to genistein (E2) (10^−6^ M) or vehicle (ethanol alone, 0.1%) for 4 days. Although EGFP expression was not observed in vehicle-treated embryos, genistein-treated embryos exhibited EGFP expression in the telencephalon of the brain (Fig. 6).

## Discussion

In this study, we first demonstrated that high genistein concentrations caused developmental toxicity in the form of decreased heart rate, retarded hatching time, decreased body length, and mortality in zebrafish embryos. Cardiac Ca^2+^ channel is activated by beta-adrenergic receptor via protein kinase A-dependant phosphorylation of the channel (Hool 2001). Genistein is a tyrosine kinase inhibitor which can interfere with the activity of several ionic channels either by altering the modulatory phosphorylating process or by direct binding (Chiang et al. 1996). In human atrial fibers, rabbit pacemaker cells, and guinea pig cardiomyocytes, negative chronotropic action of genistein is likely due to a direct blocking of Ca^2+^ channel, instead of inhibition of tyrosine kinase activity (Chiang et al. 1996; Ma et al. 2002; Altomare et al. 2006). In our results, decreased heart rate is thought to be caused by genistein via direct blocking of Ca^2+^ channel. Genistein administration in neonatal rats from birth to postnatal day 21 resulted in decreased body weight (Lewis et al. 2003). Furthermore, a high concentration of genistein-treated minnows grew more slowly than control minnows, and Xenopus embryos failed to gastrulate at all within the 48 h period in response to genistein exposure (Ingham et al. 2004). Also, when mouse blastocysts were incubated in medium containing genistein, cell proliferation and growth were inhibited (Chan et al. 2007). These developmental toxicities shown in other reports agreed well with our results. Studies using several cell systems suggested that the mechanism of genistein-induced growth inhibition includes: regulation of cell cycle checkpoints, modulation of the transforming growth factor beta-1 signaling pathway, antiangiogenesis, and antioxidant activities (Kim et al. 1998). Genistein has been shown to inhibit protein tyrosine kinase and topoisomerase-II activity, tumor cell proliferation and differentiation (Akiyama et al. 1987; Markovits et al. 1989), and may inhibit embryonic development by altering mitochondria structure and function (Bogoliubova et al. 1999).

Checking the number of hatched embryos 37 h after treatment showed almost 100% of vehicle-treated embryos were hatched and the genistein treatment showed dose-dependent inhibition of hatching rates at this time. Because the 1 × 10^−4^ M genistein-treated embryos did not hatch during all experimental periods, we did not measure the body length of the 1 × 10^−4^ M genistein-treated embryos.

To determine whether the toxicity induced by genistein was related to the negative effects on heart rate, zebrafish embryos were exposed to isoproterenol in the presence of genistein. Although isoproterenol (10 × 10^−6^ M and 100 × 10^−6^ M) increased heart rate significantly in the presence of genistein, it could not recover genistein-induced retarded hatching time, decreased body length, and mortality. Genistein-induced toxicity may be induced not by decreased heart rate completely but by other mechanisms. However, because the zebrafish embryo heart at 24 h post-fertilization doesn't respond to adrenergic stimulation completely, isoproterenol could not recover decreased heart rate until the zebrafish embryo heart responded to adrenergic stimulation completely (Milan et al. 2003; Schwerte et al. 2006).

The chronic oral treatment of rats with high doses of genistein increased lactate dehydrogenase (LDH) activity and loss of mitochondria function in rat brain tissue homogenates. Furthermore, DNA fragmentation was detected in these brain tissue homogenates (Choi and Lee 2004). In our case, we also observed apoptotic DNA fragments in the brain and spinal cord of genistein-treated embryos via TUNEL assay. This result showed that a zebrafish system can represent a specific toxic response in a genistein-treated mammalian system, and might be used to test developmental toxicity of endocrine disruptors. We also observed granular degeneration of myocytes. The morphologic changes such as kyphosis observed in genistein-treated embryos might be caused by degeneration of myocytes and neural cell apoptosis.

In addition to offering sensitivity for toxic chemicals and transparency, zebrafish embryos provide a model system that mimics embryos under intrauterine conditions. Genistein in soybean-consuming pregnant women may affect embryo development. Teratogenic effects of genistein observed in this study emphasize the risk of genistein to embryos under intrauterine conditions.

EGFP was expressed in response to genistein (10^−6^ M) on mosaic reporter zebrafish embryos. EGFP expression was localized in the telencephalon of the brain, which was not related with areas of apoptosis induced by 1 × 10^−4^ M genistein. When determining the estrogenic potential of some chemicals, in vivo assays are necessary because they can reflect in vivo functions such as absorption, metabolism, and secretion. Recently, our laboratory has developed a transgenic reporter mosaic zebrafish system for the rapid screening of chemicals for estrogenic potential. In this study, we confirmed the estrogenic potential of genistein on our transgenic reporter mosaic zebrafish system, which might be used to detect endocrine disruptors effectively.

In summary, although low doses of genistein maybe beneficial to prevent some cancers, excessive genistein might cause teratogenic effects in exposed zebrafish embryos. We also confirmed the estrogenic potential of genistein on our mosaic reporter zebrafish embryos.
